# Manipulation of Plastidial Protein Quality Control Components as a New Strategy to Improve Carotenoid Contents in Tomato Fruit

**DOI:** 10.3389/fpls.2019.01071

**Published:** 2019-09-05

**Authors:** Lucio D’Andrea, Manuel Rodriguez-Concepcion

**Affiliations:** ^1^Centre for Research in Agricultural Genomics (CRAG) CSIC-IRTA-UAB-UB, Barcelona, Spain; ^2^Consejo Superior de Investigaciones Científicas (CSIC), Barcelona, Spain

**Keywords:** carotenoids, Clp protease, chaperones, Hsp70, protein quality control, tomato

## Abstract

Carotenoids such as β-carotene (pro-vitamin A) and lycopene accumulate at high levels during tomato (*Solanum lycopersicum* L.) fruit ripening, contributing to the characteristic color and nutritional quality of ripe tomatoes. Besides their role as pigments in chromoplast-harboring tissues such as ripe fruits, carotenoids are important for photosynthesis and photoprotection in the chloroplasts of photosynthetic tissues. Interestingly, recent work in *Arabidopsis thaliana* (L.) Heynh. has unveiled a critical role of chloroplast protein quality control components in the regulation of carotenoid biosynthesis. The accumulation (i.e. degradation rate) and activity (i.e. folding status) of phytoene synthase (PSY) and other *Arabidopsis* biosynthetic enzymes is modulated by chaperones such as Orange (OR) and Hsp70 in coordination with the stromal Clp protease complex. OR and Clp protease were recently shown to also influence PSY stability and carotenoid accumulation in tomato. Here we show how manipulating the levels of plastid-localized Hsp70 in transgenic tomato plants can also impact the accumulation of carotenoids in ripe fruit. The resulting carotenoid profile and chromoplast ultrastructure, however, are different from those obtained in tomatoes from transgenic lines with increased OR activity. These results suggest that different chaperone families target different processes related to carotenoid metabolism and accumulation during tomato ripening. We further discuss other possible targets for future manipulation in tomato based on the knowledge acquired in *Arabidopsis*.

## Introduction

**Figure 1:**
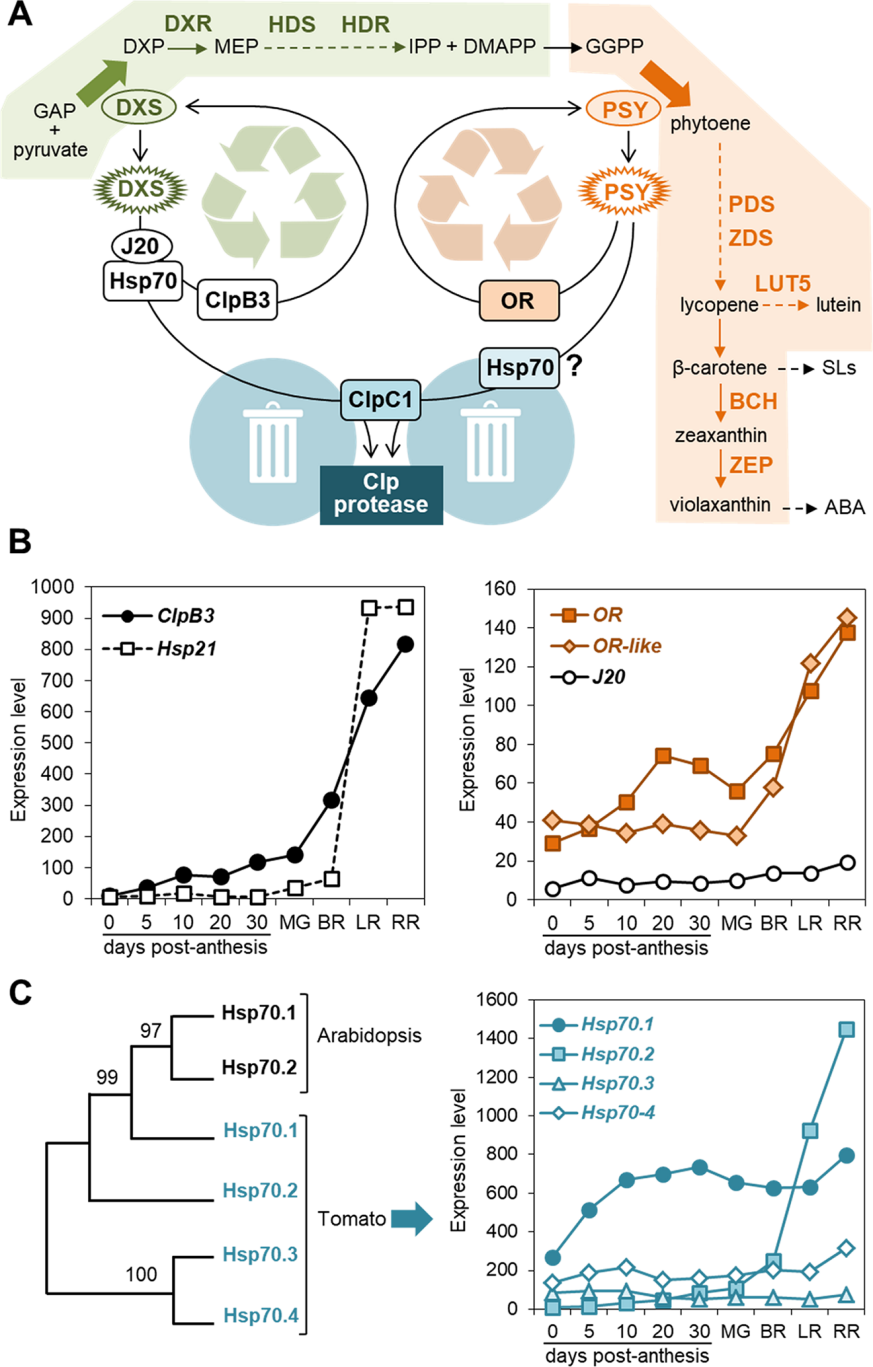
PQC components involved in carotenoid biosynthesis. **(A)** Model of the regulation of biosynthetic enzymes by PQC components. Metabolite acronyms: GAP, glyceraldehyde 3-phosphate; DXP, deoxyxylulose 5-phosphate; MEP, methylerythritol 4-phosphate; IPP, isopentenyl diphosphate; DMAPP, dimethylallyl diphosphate; GGPP, geranylgeranyl diphosphate; SLs, strigolactones; ABA, abscisic acid. Dashed arrows represent several steps. The MEP pathway (boxed in green) provides substrates for the carotenoid pathway (boxed in orange). DXS and PSY catalyze the main flux-controlling steps of these pathways (large arrows). When active enzymes (oval) misfold or/and aggregate (spiked), specific chaperones participate in their unfolding for either reactivation or removal. DXS and PSY are degraded by the stromal Clp protease, a complex that also appears to control the levels of the indicated enzymes (bold): DXR, DXP reductoisomerase; HDS, hydroxymethylbutenyl diphosphate (HMBPP) synthase; HDR, HMBPP reductase; PDS, phytoene desaturase; ZDS, zeta-carotene desaturase; LUT5, cytochrome P450 beta-hydroxylase; BCH, non-heme di-iron beta-hydroxylase; ZEP, zeaxanthin epoxidase. **(B)** Expression levels of the indicated chaperone genes during tomato fruit development. Data from the Tomato Expression Atlas (http://tea.solgenomics.net/) were plotted to show the levels of transcripts encoding ClpB3 (Solyc02g088610), Hsp21 (Solyc03g082420), OR (Solyc03g093830), OR-like (Solyc09g010110) and J20 (Solyc05g053760) in fruit at several stages after anthesis. MG, mature green (equatorial pericarp); BR, breaker (equatorial pericarp); LR, light red (total pericarp); RR, red ripe (total pericarp). **(C)** Plastidial Hsp70 family members and their expression levels during tomato fruit development. The left panel shows a Maximum Likelihood unrooted tree constructed with MEGA6 using Arabidopsis Hsp70.1 (At4g24280) and Hsp70.2 (At5g49910) and tomato Hps70.1 (Solyc01g103450), Hsp70.2 (Solyc11g020040), Hsp70.3 (Solyc01g106260), and Hsp70.4 (Solyc01g106210) protein sequences lacking their predicted plastid-targeting sequences (http://www.cbs.dtu.dk/services/TargetP/). The graph on the right was made as described in **(B)**.

Carotenoids are a group of isoprenoid molecules produced by all photosynthetic organisms and some non-photosynthetic bacteria and fungi (Rodriguez-Concepcion et al., 2018). In plants they are essential as photoprotective pigments for photosynthesis and as precursors for the production of hormones (abscisic acid, strigolactones) and other signaling molecules. They also provide colors and aromas to many flowers and ripe fruits. Dietary carotenoids and their cleavage products contribute to human and animal nutrition at many different levels. These features translate into huge economic value for the industry (e.g. as natural pigments) and major nutritional relevance for consumers (Rodriguez-Concepcion et al., 2018; Ilahy et al., 2019). In plants, carotenoids are synthesized and accumulated in plastids. They derive from geranylgeranyl diphosphate (GGPP), generated from prenyl diphosphate substrates synthetized by the methylerythritol 4-phosphate (MEP) pathway (**Figure 1A**). The metabolic flux through the MEP pathway and hence the supply of isoprenoid precursors for carotenoid biosynthesis is mainly limited by the activity of the first enzyme, deoxyxylulose 5-phosphate synthase (DXS). Another major regulatory checkpoint is the production of phytoene from GGPP catalyzed by phytoene synthase (PSY) in the first committed step of the carotenoid pathway (Camagna et al., 2019). The subsequent action of desaturases and isomerases transforms uncolored phytoene into red-colored lycopene. Cyclization of lycopene ends branches the pathway to produce β-carotene (pro-vitamin A) and derived β,β-xanthophylls (including zeaxanthin and violaxanthin) in one branch and β,ε-xanthophylls (e.g. lutein) in the other (**Figure 1A**).

Besides biosynthesis (and degradation) rates, carotenoid storage capacity is a major determinant of carotenoid contents in plant tissues (Llorente et al., 2017; Sun et al., 2018). Chromoplasts are plastids specialized in the accumulation of high carotenoid levels. Ultrastructure of chromoplasts is mainly determined by their carotenoid profiles, which widely differ among plant species, organs, and developmental stages (Sun et al., 2018). During tomato (*Solanum lycopersicum* L.) fruit ripening, chromoplasts differentiate from chloroplasts as chlorophylls degrade and carotenoids accumulate to change the fruit color from green to orange and red when ripe. The carotenoid profile also changes from typical chloroplast carotenoids (lutein, β-carotene and β,β-xanthophylls) to mainly lycopene and some β-carotene in chromoplasts (D’Andrea et al., 2018; Yazdani et al., 2019). Extensive genetic evidence indicates that increasing carotenoid sink capacity leads to enhanced levels of these health-promoting pigments in tomato fruit and other plant systems (Sun et al., 2018). Yet our limited knowledge of how chromoplast differentiation and carotenoid storage are regulated has prevented to fully exploit this “pull” strategy as an alternative or a complement to the “push” approach based on stimulating carotenoid biosynthesis.

Orange (OR) is one of the very few proteins known to influence chromoplast differentiation (Rodriguez-Concepcion et al., 2018; Sun et al., 2018). OR is a DNAJ-related protein that shows chaperone activity (Park et al., 2016). Different OR versions promote PSY accumulation and activity and some prevent carotenoid (particularly β-carotene) metabolism (Bai et al., 2014; Tzuri et al., 2015; Yuan et al., 2015; Zhou et al., 2015; Bai et al., 2016; Park et al., 2016; Chayut et al., 2017; Kim et al., 2018). In melon (*Cucumis melo* L.), a form of OR harboring an amino acid change from Arg to His at a position identified as a “golden SNP” is responsible for the high β-carotene content of some cultivars (Chayut et al., 2017). This variant (OR-His) was found to bind and promote PSY stability similarly to the OR-Arg form while being much more efficient in preventing the conversion of β-carotene into downstream products. Overexpression of *Arabidopsis thaliana* (L.) Heynh. OR-Arg and OR-His versions in tomato was recently reported to result in increased levels of fruit carotenoids (Yazdani et al., 2019). The chloroplast to chromoplast transition in transgenic fruit, which was most efficiently promoted by OR-His, was associated with increased expression of other chaperone-encoding genes (Yazdani et al., 2019). Levels of plastidial chaperones (often referred to as heat-shock proteins) actually increase during natural fruit ripening in tomato (**Figures 1B, C**) and several other plant systems, presumably to deal with proteome changes and protein folding stress resulting from the chromoplast differentiation process (Bonk et al., 1996; Neta-Sharir et al., 2005; Barsan et al., 2012; Suzuki et al., 2015; Shukla et al., 2017; D’Andrea et al., 2018). Interestingly, constitutive overexpression of one of such chaperones, small heat shock protein 21 (Hsp21), appeared to promote the conversion of chloroplasts to chromoplasts during tomato fruit ripening (Neta-Sharir et al., 2005).

Besides contributing to proper protein folding, assembly, and subcellular targeting, chaperones, together with proteases, are central components of the protein quality control (PQC) systems that ensure the stabilization, repair, or degradation of proteins that lose their native conformation and activity after stress episodes such as excess light, temperature peaks, oxidative stress or nutrient starvation (Trösch et al,. 2015; Majsec et al., 2017). They hence promote correct protein folding, remove irreversibly damaged proteins, and ensure plastid proteostasis. In agreement with the conclusion that PQC components are major regulators of fruit carotenoid accumulation and chromoplast differentiation in tomato, down-regulation of the activity of the Clp proteolytic complex (the main stromal protease) resulted in defective differentiation of fruit chromoplasts and an altered carotenoid profile during tomato ripening (D’Andrea et al., 2018). Here we revise recent advances in our understanding of the connections between PQC and carotenoid accumulation and discuss how interference with PQC components can improve the carotenoid profile of tomato fruits, one of the most highly consumed vegetables worldwide.

### Learning From *Arabidopsis*

A direct connection between PQC and carotenoid biosynthesis is well established in *Arabidopsis* (**Figure 1A**). DXS is prone to lose its native structure, resulting in protein misfolding and aggregation in chloroplasts (Pulido et al., 2013). Delivery of such inactive forms of DXS to the Hsp70 chaperone for unfolding is facilitated by their direct binding to J20, a DNAJ co-chaperone that functions as a Hsp70 adaptor (Pulido et al., 2013). The fate of Hsp70-bound enzymes depends on the relative abundance of two plastidial Hsp100 chaperones: ClpB3 and ClpC1 (Pulido et al., 2016). Under conditions promoting protein folding stress (e.g. heat), ClpB3 chaperones accumulate and assist Hsp70 to unfold DXS and release it to the chloroplast stroma, allowing spontaneous refolding to its active form. The default pathway, however, involves degradation mediated by ClpC1, a Clp protease component (Pulido et al., 2016). ClpC1 is part of the chaperone ring that unfolds protein clients prior to degradation by the proteolytic core of the complex. Both pathways (refolding and degradation) are interconnected by a mechanism involving the heat shock transcription factor HsfA2, which is induced by an unknown signal released by chloroplasts when Clp protease activity is compromised (Llamas et al., 2017). Higher HsfA2 levels induce the expression of genes encoding plastid-targeted chaperones such as ClpB3 and Hsp21, hence boosting the unfolding and disaggregation capacity of chloroplasts to cope with protein folding stress (Llamas et al., 2017). This sort of coordination between proteases and chaperones is a widespread mechanism that ensures protein homeostasis in several plant cell compartments.

Besides DXS, the Clp protease regulates the levels of other MEP pathway enzymes as well as many of the carotenoid pathway enzymes, including PSY (Rodriguez-Concepcion et al., 2019). The common regulation of both pathways by the Clp protease likely adjusts the supply of metabolic precursors for the production of carotenoids (**Figure 1A**). As described for DXS, degradation of *Arabidopsis* PSY by the Clp proteolytic complex involves interaction with ClpC1 (Welsch et al., 2018). The delivery pathway, however, appears to differ between these enzymes. PSY interacts with OR and Hsp70 chaperones (Zhou et al., 2015; Park et al., 2016; Welsch et al., 2018). Unlike J20 (a canonical DNAJ protein), OR is a DNAJE1-type protein that lacks the J-domain and hence it is unable to interact with Hsp70 (Lu et al., 2006; Pulido and Leister, 2018; Welsch et al., 2018). Consistent with the conclusion that J20 and OR play distinct roles in regulating the stability of their protein clients, higher DXS protein but lower activity levels were observed in J20-defective mutants (Pulido et al., 2013; Pulido et al., 2016) whereas higher levels of both PSY protein and activity were detected in OR-overexpressing lines (Zhou et al., 2015; Park et al., 2016; Welsch et al., 2018). The current model proposes that OR might promote refolding of misfolded or aggregated PSY proteins which otherwise would be degraded by the Clp protease by a pathway involving ClpC1 and, perhaps, Hsp70 (**Figure 1A**). In agreement with this model, PSY protein levels are up-regulated in mutants lacking Hsp70.2, one of the two Hsp70 isoforms found in *Arabidopsis* chloroplasts (Welsch et al., 2018).

### Translating to Tomato

Tomato has risen to the podium of model plants in carotenoid biotechnology due to the high abundance of carotenoids in the fruit together with the astounding genetic resources available and the recent publication of its genome sequence. From early reverse genetic approaches to manipulate the carotenoid content of tomato fruits it was deduced that post-transcriptional mechanisms are key to regulate carotenoid accumulation during fruit ripening (Giuliano et al., 2008; Fraser et al., 2009; Rodriguez-Concepcion et al., 2018). However, the specific nature of such mechanisms has remained little explored. Work in *Arabidopsis* has recently provided important clues on the relevance of PQC for this matter as well as molecular tools to manipulate the differentiation of chromoplasts and the carotenoid profile of tomato fruits. A general conclusion is that the basic mechanisms unveiled in *Arabidopsis* chloroplasts appear to be conserved in tomato chromoplasts. For example, decrease of Clp protease activity during tomato ripening using an artificial microRNA (amiRNA) against one of the catalytic core subunits resulted in increased DXS and PSY protein levels in ripe fruit (D’Andrea et al., 2018), suggesting that both enzymes are Clp protease targets in *Arabidopsis* and tomato. Also similar to that observed in *Arabidopsis* (Llamas et al., 2017), reduced Clp protease activity led to upregulated expression of genes encoding plastid-targeted chaperones such as ClpB3 in transgenic tomatoes (D’Andrea et al., 2018). The two tomato genes encoding OR homologues were also upregulated in transgenic fruits with reduced Clp protease activity (D’Andrea et al., 2018). Because transgene-mediated downregulation of fruit Clp protease activity triggers an induction of nuclear genes encoding plastid-targeted chaperones, it is possible that naturally occurring defects in the Clp protease-dependent degradation pathway during the differentiation of tomato fruit chromoplasts might be similarly signaled to the nucleus to eventually ensure correct folding (i.e. activity) of key enzymes such as DXS (*via* ClpB3) and PSY (*via* OR). Genes encoding ClpB3 and OR chaperones are induced during normal fruit ripening (**Figure 1B**), supporting a role of these chaperones in keeping carotenoid biosynthetic enzymes active as chloroplast naturally differentiate into chromoplasts. It is unknown whether other ripening-induced chaperones that protect against protein misfolding and aggregation such as Hsp21 (**Figure 1B**) or Hsp70 (**Figure 1C**) are also up-regulated in tomato as they are in *Arabidopsis* when Clp protease activity is blocked (Llamas et al., 2017).

As indicated above, both Hsp21 and OR chaperones were found to promote the conversion of chloroplasts to chromoplasts and the accumulation of carotenoids when overproduced in tomato fruit (Neta-Sharir et al., 2005; Yazdani et al., 2019). From the rest of chaperones identified in *Arabidopsis* to play a role in the carotenoid-related PQC network (**Figure 1A**), only the tomato homologues of J20 and ClpB3 have been investigated by altering their levels in transgenic plants. Their role on fruit carotenoid biosynthesis, however, was not addressed. Tomato antisense plants with reduced *J20* or *ClpB3* transcript levels showed reduced thermotolerance (Yang et al., 2006; Wang et al., 2019). By contrast, transgenic tomato lines overexpressing the endogenous *J20* gene showed enhanced thermotolerance, in part because the expression of *HsfA2* and other genes encoding heat shock transcription factors was higher in *J20*-overexpressing lines under heat stress (Wang et al., 2019). Interestingly, the J20 homologue was found to interact with Hsp70.2 (Wang et al., 2019), one of the four plastid-targeted isoforms of Hsp70 present in tomato (**Figure 1C**). *Hsp70.2* (but not *J20* or other Hsp70-encoding genes) is strongly upregulated during fruit ripening (**Figure 1C**). Hsp70.2 also interacts with the J20-related DNAJ proteins CDJ1 and CDJ2, which maintain photosystem II under chilling stress (Kong et al., 2014) and protect Rubisco from degradation under heat stress (Wang et al., 2015). No functional information is available for the other three tomato plastid-targeted Hsp70 isoforms.

### Filling the Gaps

**Figure 2:**
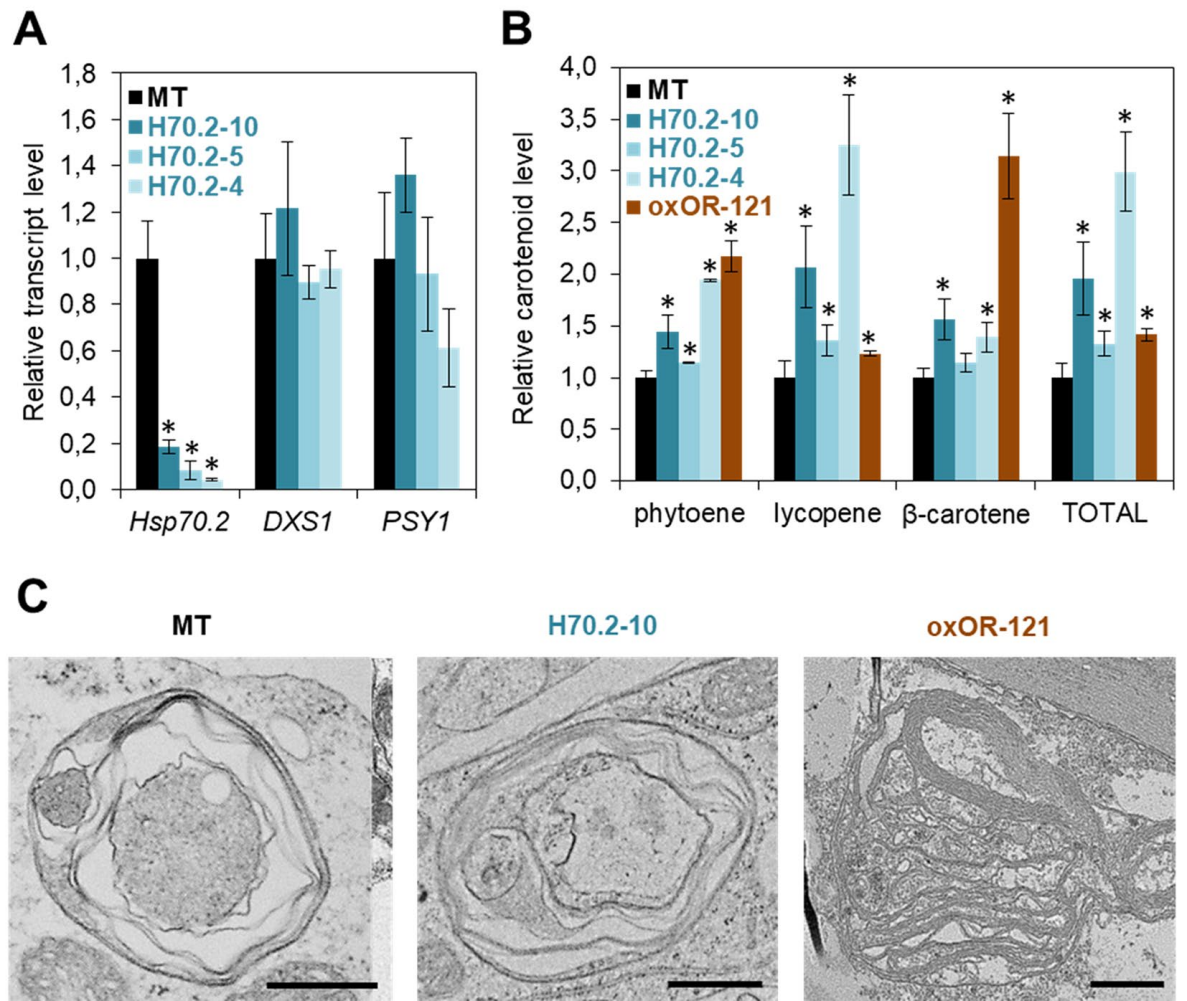
Hsp70 and OR chaperones differentially control carotenoid biosynthesis in tomato. **(A)** Transcript levels of the indicated genes in the pericarp of red ripe fruits. Tomato MicroTom (MT) plants were transformed with an amiRNA construct to silence the *Hsp70.2* gene during fruit ripening. Ripe fruits from greenhouse-grown untransformed and transgenic T1 plants were collected at 52 days post-anthesis (dpa) and used for real-time quantitative PCR (qPCR) analysis of *Hsp70.2*, *DXS1* (Solyc01g067890) and *PSY1* (Solyc03g031860) expression using the *ACT* gene (Solyc04g011500) as normalizer. Mean and SD values (n ≥ 2) are shown relative to those in untransformed MT fruit. Asterisks mark statistically significant differences relative to MT samples (T-test, *P* < 0.05). **(B)** Levels of carotenoids in the pericarp of red ripe fruits from the indicated lines. MT plants were transformed with a construct previously used to constitutively overexpress Arabidopsis OR-His under the control of the *35S* promoter in tomato plants of the M82 cultivar (Yazdani et al., 2019). A representative T1 line was selected based on the distinctive orange color of their fruit. T2 plants of this line (oxOR-121) were grown in the greenhouse together with untransformed controls and T2 plants of *Hsp70.2*-silenced lines analyzed in **(A)**, and ripe fruits were collected at 52 dpa for pericarp carotenoid extraction and analysis by HPLC. Mean and SD values (n ≥ 2) of individual and total carotenoid levels are shown relative to those in MT fruit. Asterisks mark statistically significant differences relative to MT samples (T-test, *P* < 0.05). **(C)** Representative images of chromoplasts. Fruits were collected from the indicated lines at 52 dpa and used for TEM analysis of chromoplast ultrastructure. Bar, 1 µm. Previously described methods (D’Andrea et al., 2018) were used for amiRNA synthesis, plant transformation, RNA and carotenoid extraction, qPCR, HPLC, TEM, and statistical analyses.

Hsp70 chaperones appear as a central node in the *Arabidopsis* carotenoid-related PQC network (**Figure 1A**) but their role in tomato ripening has not been addressed. To fill this gap, we generated transgenic tomato plants with decreased expression of the ripening-induced *Hsp70.2* gene (**Figure 2**). Constructs were generated as described (D’Andrea et al., 2018) to express a *Hsp70.2*-specific amiRNA under the control of the ripening-induced *PLI* promoter (Estornell et al., 2009). After transformation of tomato cv. MicroTom (MT) plants, T1 lines with decreased levels of *Hsp70.2* transcripts were selected for further studies. The fruits of these lines showed no significant changes in the expression of tomato genes encoding the fruit DXS1 and PSY1 isoforms (**Figure 2A**). Ripe fruits from T2 lines showed increased levels of lycopene and, to a lower extent, other fruit carotenoids (**Figure 2B**). Consistent with their high lycopene enrichment, transgenic fruit of the H70.2-10 line showed a stronger red color. These results suggest that Hsp70.2 negatively regulates carotenoid biosynthesis. This role might involve delivering fruit PSY1 and likely other carotenoid pathway enzymes to degradation by the Clp protease complex (**Figure 1A**). Protein complexes containing Hsp70 and enzymatically inactive forms of phytoene desaturase (PDS in **Figure 1A**), a possible Clp protease client (Welsch et al., 2018; Rodriguez-Concepcion et al., 2019), have actually been found in chromoplasts (Al-Babili et al., 1996; Bonk et al., 1996). Loss of Hsp70.2 might make inactive enzymes more available for reactivation upon unfolding by chaperones such as OR in the case of PSY1. While it is possible that Hsp70.2 might regulate other processes related to carotenoid biosynthesis or storage, both ripe fruit color and chromoplast ultrastructure were similar in MT and Hsp70.2-defective lines (**Figure 2C**). Thus, chromoplasts from transgenic ripe fruits contained similar vesicles, plastoglobules, and undulating membranes (i.e. the remnants of envelopes formed around lycopene crystals) as those observed in untransformed controls (**Figure 2C**). It is possible that other plastidial Hsp70 chaperones besides Hsp70.2 might ensure protein homeostasis during chromoplast differentiation in Hsp70.2-defective transgenic lines. At least some of them might also ensure that the tomato DXS1 enzyme also remains active to provide more substrates for PSY1 to channel into the carotenoid pathway during fruit ripening.

Consistent with the conclusion that Hsp70 and OR have opposite roles, tomato fruit carotenoids can be increased either by down-regulating Hsp70 (**Figure 2**) or by up-regulating OR (Yazdani et al., 2019). But unlike Hsp70.2-defective lines, constitutive overexpression of the *Arabidopsis* OR-His protein in tomato MT plants produced fruits that were most enriched in phytoene (likely the result of enhanced PSY activity) and β-carotene instead of lycopene (**Figure 2B**). The enrichment in β-carotene, which resulted in ripe fruits with a distinctive orange hue in some lines is similar to that reported in other plant systems when OR-His proteins are overproduced. It has been suggested that OR might repress β-carotene metabolism or/and degradation by interacting with cleaving enzymes such as CCD4 (Chayut et al., 2017; Kim et al., 2018). Also similar to other plant systems (Yuan et al., 2015; Park et al., 2016), OR-His promoted the development of membranous chromoplasts in tomato ripe fruit (**Figure 2C**). The available data allow to conclude that Hsp70 and OR play distinct but complementary roles for both carotenoid biosynthesis (i.e. enzyme activity) and storage (i.e. chromoplast differentiation) during normal fruit ripening in tomato.

### Looking Forward

The described results demonstrate that manipulation of plastidial PQC components related to carotenoid metabolism and storage works to modify the carotenoid profile of tomato fruits. Manipulation of Hsp21, OR or/and Clp protease activity has additionally been found to increase tolerance to environmental stress (Neta-Sharir et al., 2005; D’Andrea et al., 2018; Kim et al., 2018). PQC components identified in *Arabidopsis* to regulate carotenoid biosynthesis and found in tomato to improve thermotolerance, such as J20 (Wang et al., 2019), ClpB3 (Yang et al., 2006) or HsfA2 (Fragkostefanakis et al., 2016), remain unexplored in terms of their impact on tomato fruit carotenoid accumulation. We hypothesize that the overexpression of ClpB3 might lead to higher levels of carotenoids but also other MEP-derived plastidial isoprenoids such as tocopherols (vitamin E) due to a predicted higher DXS activity (Llamas et al., 2017). As ClpB3 also promotes the disaggregation, assembly and stability of other plastidial proteins and complexes (Lee et al., 2007; Mishra and Grover, 2016), its overproduction might provide additional benefits to fruit ripening, including an enhanced resistance to multiple stresses affecting protein folding. A similar but maybe enhanced phenotype might be achieved by the ripening-specific overexpression of the transcription factor HsfA2, found to upregulate the expression of genes encoding chaperones such as ClpB3 and Hsp21 in *Arabidopsis*. Future research should also identify the motifs in carotenoid biosynthetic enzymes that determine their misfolding propensity or stability as well as the basic mechanisms underlying chromoplast differentiation. Such information would provide new powerful tools to improve the nutritional quality of tomatoes and other fruits.

## Data Availability

The datasets generated for this study are available on request to the corresponding author.

## Author Contributions

LD’A performed experiments and collected data and information. LD’A and MR-C analyzed the data, discussed the results, prepared the figures, and wrote the paper.

## Funding

This work was supported by grants from the Spanish Ministry of Economy and Competitiveness (BIO2017-84041-P and BIO2017-90877-REDT), the Ministry of Education, Culture and Sports (AP2012-0189), the Generalitat de Catalunya (2017SGR-710), and the European COST Action EuroCaroten (CA15136). We also thank the financial support of the MINECO Severo Ochoa Programme for Centres of Excellence in R&D 2016-2019 (SEV-2015-0533) and the Generalitat de Catalunya CERCA Programme to CRAG.

## Conflict of Interest Statement

The authors declare that the research was conducted in the absence of any commercial or financial relationships that could be construed as a potential conflict of interest.
